# Knowledge, risk perception, and prevention preparedness towards COVID-19 among a cross-section of animal health professionals in Nigeria

**DOI:** 10.11604/pamj.2022.41.20.28315

**Published:** 2022-01-09

**Authors:** Ismail Ayoade Odetokun, Nma Bida Alhaji, Uduak Akpabio, Madinat Abimbola Abdulkareem, Gladys Taye Bilat, Deepak Subedi, Ibrahim Ghali-Mohammed, Nusirat Elelu

**Affiliations:** 1 Department of Veterinary Public Health and Preventive Medicine, University of Ilorin, Ilorin, Kwara State, Nigeria,; 2 Department of Public Health and Epidemiology, Niger State Ministry of Livestock and Fisheries, Minna, Niger State, Nigeria,; 3 Department of Veterinary Public Health and Preventive Medicine, Michael Okpara University of Agriculture, Umudike, Nigeria,; 4 Department of Regional Disease Surveillance System Enhancement (REDISSE), Ministry of Agriculture and Food Security, Ekiti State, Nigeria,; 5 Institute of Agriculture and Animal Science (IAAS), Paklihawa campus, Tribhuvan University, Kirtipur 44618, Nepal

**Keywords:** COVID-19, knowledge, risk perception, animal health professionals, Nigeria

## Abstract

**Introduction:**

the on-going COVID-19 pandemic caused by the SARS-CoV-2 virus has imposed serious public health and economic threats on the entire world population. The SARS-CoV-2 has been identified from both domestic and wild animals constituting a threat to humans since most apparently healthy animals may potentially infect and cause the disease in humans, especially Animal Health Professionals (AHPs) who come directly in contact with animals. These professionals such as veterinarians play important roles among the One Health team in controlling the pandemic. This survey was conducted to assess the knowledge, risk perception, and prevention behaviors of AHPs in Nigeria who are important personnel in the control of emerging and re-emerging zoonotic and infectious diseases.

**Methods:**

an online-based epidemiological cross-sectional pre-tested questionnaire survey was carried out from April to May 2020. A total of 427 AHPs joined this survey. Descriptive statistics, chi-square test, and binary logistic regression model were used to analyze the data collected.

**Results:**

the respondents have a mean knowledge score of 7.34 ± 2.05 (from a total possible score of 11) with the majority (66.7%, n = 285) having satisfactory knowledge scores on COVID-19. A high proportion (240/353) of respondents reported bats to be the major wildlife incriminated in the transmission of the COVID-19 virus though other animals reported were tiger, monkey, lion, and pangolin. At least, a quarter 131 (30.7%) of respondents perceived that visiting live animal markets is of high zoonotic risk to the spread of coronavirus. Most respondents reported hands washing and sanitizing after handling animals, and using personal protective equipment when handling suspected animals. At p<0.05; respondents' age, marital status, professional status, and geopolitical zone were significantly linked with satisfactory knowledge. Veterinarians (OR=0.40; 95%CI: 0.22–0.75) were thrice less likely to possess unsatisfactory knowledge of COVID-19 than laboratory technologists.

**Conclusion:**

participants in this survey have a satisfactory level of COVID-19 knowledge and good mitigation measures instituted while working at their stations. However, AHPs need more enlightenment about the various zoonotic risk pathway contributing to the transmission of COVID-19.

## Introduction

A highly infectious and newly emerging respiratory coronavirus disease (COVID-19) was reported among humans during the last month of 2019 in China [1-3]. The disease was first named a novel coronavirus disease but was subsequently confirmed as the severe acute respiratory syndrome coronavirus 2 (SARS-CoV-2), a single-stranded RNA virus of the family *Coronaviridae*. From the foremost account of the defined case of the COVID-19 in Wuhan, China [2], the world has experienced huge mortality and morbidity due to this highly transmissible disease causing serious community health crises and colossal human impacts globally. This disease has imposed serious public health and economic threats on the entire world population [4]. COVID-19 shares similarity with the previously emerged Severe Acute Respiratory Syndrome Coronavirus (SARS-CoV) and the Middle East Respiratory Syndrome Coronavirus (MERS-CoV) [5] but has taken a devastating pandemic pattern with two spikes witnessed within a year. Currently, at least 170 million confirmed cases and 3.5 million deaths have been reported worldwide [6]. In West Africa, Nigeria currently (as of 30 May 2021) precedes the number of confirmed cases (166,285) and mortalities (2017) due to SARS-CoV-2 [7] with no indication that the curve would be flattened soon, despite measures instituted against the disease.

Although it has not been proven that SARS-CoV-2 originated from wild bats, most of the humans affected by SARS-CoV-2 in Wuhan, China have been stated to have links with seafood and animal markets, indicating a possible zoonotic spread of the virus from animals [8,9]. The virus has been identified in some animals such as domestic, pet, and wild animals [10,11], and cats were found as the most susceptible pets to the infection [12]. Also, the virus has been recovered from some environmental samples [13]. COVID-19 is regarded as a complex emerging infectious disease whose spread is possibly driven by increasing demand for food especially meat from wild animals in more resource-demanding communities [14]. This virus constitutes a threat to humans since most apparently healthy animals may potentially infect and cause the disease in humans, especially, AHPs who come directly in contact with animals. Thus, the One Health concept is useful in understanding the spread, prevention, and control of COVID-19 [2,9,14]. Animal health professionals such as veterinarians play important roles among the One Health team in controlling the pandemic [15]. During this pandemic, AHPs are regarded as essential workers rendering animal healthcare services to communal inhabitants during compulsory stay indoor policy (lockdowns) [16]. Also, they partake in key activities to ensure sustainability in food protection, disease prevention, and emergency management while maintaining public health [17].

With the current second wave and daily increase in the number of morbidity and mortality of the pandemic observed, Africa may become a major epicenter of the virus if not prevented. For instance, the COVID-19 infection is still recorded daily across Nigeria with all the 36 States and the Federal Capital Territory affected despite the fact that some vaccines have been administered to the public. Some AHPs have been drafted as members of the State and National Task Forces instituted to stop this pandemic. The risk of exposure to COVID-19 among animal health workers and the public comes from contacts among people [16]. Any deficit observed among AHPs when delivering healthcare services could compromise efforts established to control the pandemic, since perception and concerns about COVID-19 could vary among workers. It is therefore imperative to understand their knowledge levels and attitude towards the disease. This study is guided by the health Belief Model (HBM) [18] useful as a guiding background in studying the individual perception of disease risk and personal intervention to mitigate the risk [19-21]. In the HBM, people tend to engage in preventative actions when they perceive the health threat to be serious, feel susceptible to the health event, and most likely get a positive benefit from adopting a practice [22]. Individual knowledge, behaviors, and risk perception levels affect the way an individual will adhere to recommended infection control and prevention practices against a disease [23]. A change in behavior of animal health workers could influence the risk of COVID-19 spread and undermine One Health solutions to the disease. To inform animal health workers about effectual first-line public health and protective actions on zoonoses, understanding the infectious disease protective behavior among AHPs is important [23]. This survey, therefore, determines the knowledge, risk perception, and preventive preparedness towards COVID-19 among AHPs in Nigeria.

## Methods

**Study design, area, and population:** an epidemiological electronic-based pre-tested questionnaire survey was carried out from 26^th^ April to 16^th^ May 2020. The targeted respondents are the AHPs (veterinarians, para-veterinarians, and laboratory technologists) across the six geo-political zones (Northcentral, Northeast, Northwest, Southsouth, Southeast, and Southwest) which make up the 36 States (including the Federal Capital Territory) in Nigeria. All the states were sampled. At present, Nigeria ranks highest in the confirmed number of morbidity and mortality attributed to the COVID-19 pandemic in the West African sub-region and ranks 8^th^ in Africa. Nigeria is the world´s most populous black nation with approximately 206,139,589 inhabitants [7] and an abundance of animal and environmental resources. Animal health professionals that are at least 17 years of age and with a minimum of one year of work experience were called to participate in the survey. Those exempted from this study were the non-animal health workers.

**Sample size and sampling:** in this study, the sample size was computed employing the Open Source Epidemiologic Statistics for Public Health (OpenEpi), v.3.01 (updated 2013/04/06) [24]. A sample size of 422 was obtained based on the assumption that the frequency of the outcome variable of interest (knowledge) in the population is 50%, an absolute precision at 95% confidence, a 5% level of error, and a design effect of 1. This gave a computed value of 384. However, 10% non-response was added to give a final least sample size of 422. The convenience sampling procedure was employed using a web-based survey tool, to reach the target population. The questionnaire was mainly shared via email and WhatsApp platforms due to the strict lockdown policy imposed throughout the country.

**Questionnaire design, pre-testing, and implementation:** the survey instrument was made using google forms (Alphabet Inc., California, USA) and harvested information on four major areas: the respondents´ demography, knowledge/awareness on COVID-19, perceptions on zoonotic risk pathways for the transmission of COVID-19, and mitigation measures instituted against COVID-19 by AHPs. In the first section of the questionnaire, the collected demographic features of respondents include age, gender, marital status, professional status, the highest level of formal education, and the respondent's geopolitical location. To determine the knowledge level of respondents on COVID-19, nine major questions were asked from participants covering COVID-19 outbreak, susceptibility in humans and animals (domestic and wild), signs and symptoms, and zoonosis. To understand respondents' perception of the zoonotic risk pathways for COVID-19, questions such as risks of visiting live animal markets, consuming infected raw meat and meat products, having contact with contaminated or infected animals and fomites, and environmental contamination with viral aerosols were asked. The last section of the questionnaire covers questions on mitigation measures utilized by animal health workers against the spread of COVID-19. These include questions on washing and sanitizing hands after handling animals, hands washing with soap before ingestion at work, disposing off used kits in appropriate containers, routinely isolating suspected animals from others, restricting human contacts with suspected animals, equipment sterilization after usage, and the practice of using personal protective equipment (PPE) before handling suspected cases. To ensure that the questionnaire is standardized and of high quality, three public health experts reviewed the survey tool for content validity and a pre-test was performed on five veterinarians from Northcentral Nigeria. Feedbacks received was used to improve upon the questionnaire before administering the tool via email and WhatsApp platforms online to respondents. Administration of the questionnaire commenced on April 26, 2020, 6: 12: 57 PM GMT+1 and ended on May 16, 2020, 9: 10: 36 AM GMT+1 after the desired sample size was met. Although respondents were allowed the opportunity of voluntary participation without prejudice, they gave informed consent before completing the questionnaire while maintaining the confidentiality of data collected in adherence to the guidelines recommended by the 2013 Helsinki Declaration for Ethical principles as documented by the World Medical Association. The Research Ethics Committee, Niger State Ministry of Livestock and Fisheries Development approved the study (approval number MLFD/NGS/003/21).

**Data management and statistical analysis:** to summarize the data collected, the Microsoft Excel package, version, 2019 was used. These data were then exported into the Open Epi and the Statistical Package for the Social Sciences (SPSS) software, v.22, for further analyses. Summarization of the data was done using descriptive statistics (mean/standard deviation), and percentages/proportions for quantitative and categorical variables, respectively. To assess the level of the respondents' knowledge on COVID-19, correct and wrong responses provided in the knowledge section were scored numerically as 1 and 0, respectively, with the total presented as the outcome variable. The outcome variable (knowledge score) ranged from 3 to 11 (maximum obtainable score) with a mean of 7.34 ± 2.05. The knowledge score was classified as binary (satisfactory or unsatisfactory) centered on a cut-off point (average scores) [25-27]. Therefore, respondents considered as having satisfactory knowledge level were those with knowledge scores greater or equal to the average score. Correlation between the independent variables (demographics: age, gender, marital status, professional status, the highest level of formal education, and geopolitical location) and the outcome variable was made using the chi-square test. Significant independent variables at p < 0.05 were subjected to a binary logistic regression model.

## Results

**Demographic features of respondents:** a total of 427 AHPs across all the 36 states, plus the Federal Capital Territory, in the six geo-political zones in Nigeria, participated in this survey (Table 1). Respondents had a mean age of 36.02 ± 1.02 years (minimum: 17, maximum: 67). The majority of the participants were males (57.4%), married (61.6%), and veterinarians (68.1%) reporting their highest level of education as the first degree (47.1%).

**Table 1:** demographic features of the participants (n = 427)

Demographics	Category	Frequency (n)	Proportion (%)	Confidence interval
Age (years)	< 26	67	15.7	12.47, 19.37
	26 - 45	285	66.7	62.17, 71.09
	> 45	75	17.6	14.17, 21.39
Gender	Male	245	57.4	52.64, 62.01
	Female	181	42.4	37.76, 47.12
	Prefer not to say	1	0.2	0.01, 1.15
Marital status	Single	163	38.2	33.65, 42.85
	Married	263	61.6	56.91, 66.12
	Prefer not to say	1	0.2	0.01, 1.15
Professional status	Laboratory Technologist	47	11.0	8.29, 14.25
	Para-veterinarian	89	20.8	17.19, 24.89
	Veterinarian	291	68.1	63.61, 72.44
Educational level	Ph.D.	69	16.2	12.90, 19.88
	Masters	105	24.6	20.68, 28.84
	First degree	201	47.1	42.37, 51.82
	Diploma	37	8.7	6.27, 11.62
	High school	15	3.5	2.06, 5.60
Geopolitical zone	Northcentral	133	31.1	26.89, 35.66
	Northeast	14	3.3	1.88, 5.31
	Northwest	39	9.1	6.67, 12.15
	Southwest	86	20.1	16.54, 24.15
	Southeast	106	24.8	20.90, 29.09
	Southsouth	49	11.5	8.71, 14.77

**Figure 1:**
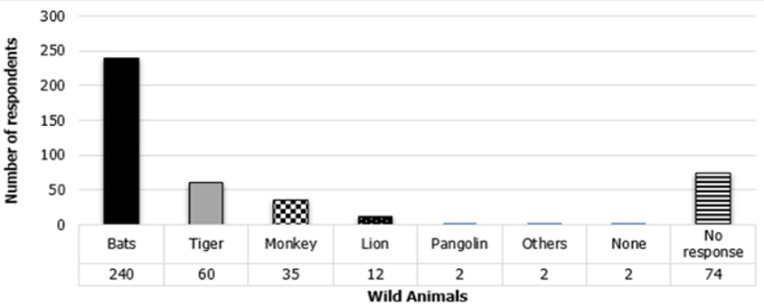
reported wild animals incriminated in the spread of COVID-19 by animal health professionals in Nigeria

**Figure 2:**
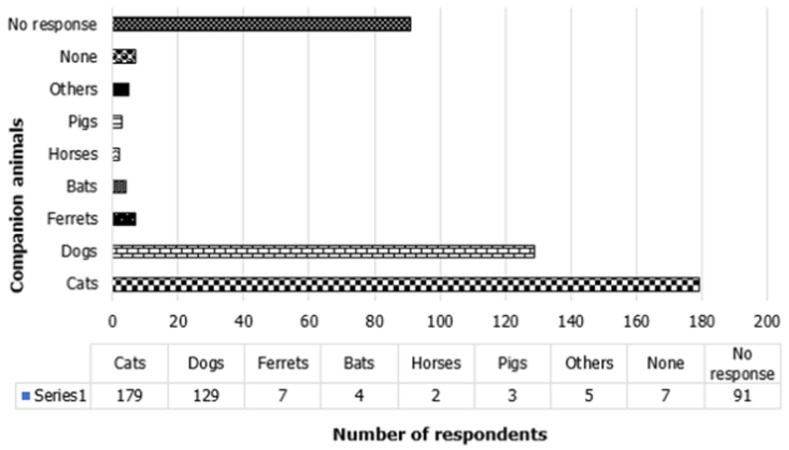
most susceptible companion animals to COVID-19 reported by animal health professionals in Nigeria

**Knowledge of COVID-19 among animal health professionals:** Table 2 includes the responses on knowledge on COVID-19 among AHPs in Nigeria. The majority of the surveyed respondents have satisfactory knowledge scores (66.7%, n = 285) on COVID-19. All the professionals (100%, n = 427) reported having heard about COVID-19 and also reported that humans and wild animals can be infected with the COVID-19 virus. A high proportion (240/353) of respondents reported bats to be the major wild animal incriminated in the COVID-19 virus transmission (Figure 1). Other animals reported to be involved in the spread of the Coronavirus are tiger (60/353 of respondents), monkey (35/353 of respondent), lion (12/353 of respondents), and pangolin (2/343 of respondents). The majority (98.4%, n = 420) reported that COVID-19 outbreaks among the human population have occurred in Nigeria though only 100 (23.4%) reported to have seen humans with clinical symptoms indicative of COVID-19. Three-quarter of the animal health workers (74.9%, n = 320) reported that companion animals can get COVID-19 virus infection. Of the 336 responding to the question on which companion animal is most susceptible to and could spread the COVID-19 virus, 179 (53.3%) and 129 (38.4%) indicated that cats and dogs were the most susceptible (Figure 2). However, only a few of the respondents (5.6%, n = 24) reported having ever seen animals with clinical signs suggestive of COVID-19 infection. The majority of AHPs reported that COVID-19 can be transmitted as a form of zoonosis from animals to humans (68.1%, n = 291) and as zooanthroponosis from humans to animals (66.3%, n = 283).

**Table 2:** knowledge, perception on zoonotic risk pathways, and mitigation practices about COVID-19 among animal health professionals in Nigeria

Question	Responses (n = 427)		
Knowledge of COVID-19	Yes n (%)	No n (%)	
Have you ever heard about COVID-19?	427 (100.0)	0 (0.0)	
Can humans be infected with COVID-19 virus?	427 (100.0)	0 (0.0)	
Has COVID-19 outbreaks occurred in Nigeria?	420 (98.4)	7 (1.6)	
Have you ever seen humans with clinical symptoms indicative of COVID-19?	100 (23.4)	327 (76.6)	
Can companion animals be infected with COVID-19 virus?	320 (74.9)	107 (25.1)	
Can wild animals be infected with COVID-19 virus?	427 (100)	0 (0.0)	
Have you ever seen animals with clinical signs indicative of COVID-19?	24 (5.6)	403 (94.4)	
Can COVID-19 be transmitted from animals to humans (zoonosis)?	291 (68.1)	136 (31.9)	
Can COVID-19 be transmitted from humans to animals (zooanthroponosis)	283 (66.3)	144 (33.7)	
Perceptions on zoonotic risk pathways	Low n (%)	Medium n (%)	High n (%)
Visiting live animal markets	132 (30.9)	164 (38.4)	131 (30.7)
Consumption of infected raw meat and meat products	60 (14.1)	145 (34.0)	222 (52.0)
Contacts with infected animals and contaminated fomites	16 (3.7)	57 (13.3)	354 (82.9)
Environmental contamination with viral aerosols	12 (2.8)	43 (10.1)	372 (87.1)
Mitigation measures	Yes n (%)	No n (%)	
Washing and sanitizing hands after handling animals	425 (99.5)	2 (0.5)	
Washing hands with soap before eating at work	426 (99.8)	1 (0.2)	
Disposing used kits in appropriate containers	425 (99.5)	2 (0.5)	
Routinely isolating suspected animals from others	424 (99.3)	3 (0.7)	
Restriction of people from having contacts with suspected animals	420 (98.4)	7 (1.6)	
Sterilization of all equipment after use	424 (99.3)	3 (0.7)	
Use of PPE (overalls/gown, gloves, cap and face mask) before handling suspected cases	426 (99.8)	1 (0.2)	

**Animal health professionals´ perception on zoonotic risk pathways of the spread of COVID-19:** at least, a quarter 131 (30.7%) perceived that visiting live animal markets is of high zoonotic risk to the spread of coronavirus (Table 2). About half 222 (52.0%) of the respondents perceived that the consumption of infected raw meat and meat products is of a high zoonotic risk pathway to the transmission of COVID-19. However, a larger proportion of the respondents to this survey (82.9% and 87.1%) regarded contact with infected animals and contaminated fomites as well as environmental contamination with viral aerosols to be related to the high zoonotic risk pathway for the spread of COVID-19.

**Risk mitigation measures by animal health professionals:** the reported mitigation measures instituted by the respondents are presented in Table 2. At least 420 (98.4%) of respondents reported washing and sanitizing their hands after handling animals, hands washing with soap before consumption of food at work, properly disposing of used kits, routine isolation of suspected animals, restricting contact with suspected animals, using PPE when handling suspected animals, and sterilizing all equipment after usage as mitigation practices they engage in on COVID-19 control.

**Factors influencing knowledge of COVID-19 among animal health professionals in Nigeria:** at p < 0.05, we found that age, marital status, professional status, and geopolitical zone were significantly associated with satisfactory knowledge of COVID-19 among respondents (Table 3). However, binary logistic regression analyses revealed that respondents within 26 - 45 years (OR = 0.45; 95% CI: 0.26 - 0.77) and > 45 years (OR = 0.31; 95% CI: 0.15 - 0.63) were twice and thrice significantly less likely to have satisfactory knowledge than respondent < 26 years while married AHPs (OR = 0.56; 95% CI: 0.37 - 0.84) were twice less expected to possess satisfactory knowledge about COVID-19. Also, veterinarians (OR = 0.40; 95% CI: 0.22 - 0.75) were thrice less likely to possess unsatisfactory knowledge of COVID-19 than laboratory technologists. Respondents from the Northwestern part of Nigeria (OR = 0.31; 95% CI: 0.12 - 0.79) were thrice less probable to be educated about COVID-19 than those from the Northcentral part of the country.

**Table 3:** factors associated with the knowledge of COVID-19 among animal health professionals in Nigeria (n = 427)

		Knowledge level					
Demographics		Satisfactory	Unsatisfactory	P-value (χ2)	OR	CI	P-value
Age (years)							
< 26	67 (15.7)	33 (49.25)	34 (50.75)	0.002*	1.00	-	-
26 - 45	285 (66.7)	195 (68.42)	90 (31.58)		0.45	0.26, 0.77	0.006*
> 45	75 (17.6)	57 (76.00)	18 (24.00)		0.31	0.15, 0.63	0.002*
Gender							
Male	245 (57.4)	160 (65.31)	85 (34.69)	0.613	-	-	-
Female	181 (42.4)	124 (68.51)	57 (31.49)		-	-	-
Prefer not to say	1 (0.2)	1 (100.00)	0 (1.00)		-	-	-
Marital status							
Single	163 (38.2)	97 (59.51)	66 (40.49)	0.014*	1.00	-	-
Married	263 (61.6)	188 (71.48)	71 (26.99)		0.56	0.37, 0.84	0.007*
Prefer not to say	1 (0.2)	1 (100.00)	0 (1.00)		-	-	-
Professional status							
Laboratory Technologist	47 (11.0)	24 (51.06)	23 (48.94)	0.002*	1.00	-	-
Para-veterinarian	89 (20.8)	51 (57.30)	38 (42.69)		0.78	0.38, 1.58	0.606
Veterinarian	291 (68.1)	210 (72.16)	81 (27.84)		0.40	0.22, 0.75	0.008*
Educational level							
Ph.D.	69 (16.2)	48 (69.57)	21 (30.43)	0.522	-	-	-
Masters	105 (24.6)	76 (72.38)	29 (27.62)		-	-	-
First degree	201 (47.1)	127 (63.18)	74 (36.82)		-	-	-
Diploma	37 (8.7)	25 (67.57)	12 (32.43)		-	-	-
High school	15 (3.5)	9 (60.00)	6 (40.00)		-	-	-
Geopolitical zone							
Northcentral	133 (31.1)	84 (63.16)	49 (36.84)	0.000*	1.00	-	-
Northeast	14 (3.3)	6 (42.86)	8 (57.14)		2.29	0.75, 6.97	0.234
Northwest	39 (9.1)	33 (84.62)	6 (15.38)		0.31	0.12, 0.79	0.016*
Southwest	86 (20.1)	45 (52.33)	41 (47.67)		1.56	0.90, 2.71	0.147
Southeast	106 (24.8)	78 (73.58)	28 (26.42)		0.61	0.35, 1.07	0.114
Southsouth	49 (11.5)	39 (79.59)	10 (20.41)		0.44	0.20, 0.96	0.050

* - significant at p < 0.05; CI - Confidence Interval; OR -Odds ratio

## Discussion

Coupled with the local spread in the initial phase of the COVID-19 outbreak, the number of cases of the virus is still increasing in Nigeria thereby making Nigeria precedes the morbidity rate and mortality attributed to the COVID-19 pandemic in the West African sub-region and ranks 8^th^ in Africa. Lagos State is the country´s epicenter for the spread of COVID-19, due to people traveling to the state from the international communities through the air, making the residents more susceptible to COVID-19 infection. The Nigerian government encompassing the Ministry of Health in collaboration with the Nigeria Center for Disease Control (NCDC) adopted strategies coined 'infection, prevention, and control (IPC)' aimed at curtailing the pandemic COVID-19 in humans. The underlying characteristics are the level of preparedness, awareness, and knowledge of the spread and prevention as shown in the aggressive campaigns by NCDC [28]. Though epidemiological studies on COVID-19 have been carried out in Nigeria among some health professionals and in some geographic regions [28-31], we present the foremost nationwide survey on the knowledge, risk perception, and preventive preparedness among animal health workers.

Our findings elucidated that the general knowledge about the COVID-19 outbreak was 66.7%. The above-average knowledge scores on COVID-19 reflects the current perception of COVID-19 among the general populace. An earlier study [26] reported a comparable knowledge level about the disease among the Nigerian population. A similar survey carried out by Serwaa *et al*. (2020) in Ghana [32] reported a general knowledge of the COVID-19 outbreak to be 61.7% while Zhong *et al*. (2020) in China recorded an overall rate of 90% [33]. This finding was more than that conducted in Ghana and lower than the one conducted in China. This difference might be due to the dissimilarity in the investigated populations. The knowledge of COVID-19 recorded might be due to the reports of the diseases from different news channels that covered the outbreak of the infection in the country.

All the professionals reported having heard about COVID-19 and also reported that humans and animals can be infected with the SARS-CoV-2 virus. COVID-19 is judged to be an evolving disease by the World Organization for Animal Health (OIE). It appears that it can be zooanthroponotic in some situations. Few numbers of animals worldwide have been stated to be infected with SARS-CoV-2 [9], mainly after intimate contact with COVID-19 infected persons, with 68 confirmed cases in cats, dogs, and minks in the United States [34].

A high proportion (67.9%) of respondents in this survey reported bats to be the major wild animal incriminated in the SARS CoV-2 virus spread. The ideal reservoir host for coronavirus is bats because the viruses inhabit the bats with no symptoms seen. Bats can spread the virus to various hosts when they fly around in search of food, especially within the forest [35]. In the current SARS CoV-2 pandemic, the virus is identified in the laboratory to be 96% identical to the bat CoV at the genomic level making bats a suspected primary source in the COVID-19 outbreaks [1,36]. Other animals reported to be involved in the spread of the Coronavirus are tiger 16.9%, monkey (9.9%), lion (3.4%), and pangolin (0.5%). Other wild species such as monkeys, pangolins, and others when they are being in contact with, can lead to the initiation and spreading of zoonoses. Humans are known to hunt wild animals for meat throughout history. Currently, bush-meat consumption is tremendously increasing in most parts of the world. It can serve as an important source of nutrients for low-earning and food-insecure households in forests and rural areas, especially in Africa [37]. Lack of regulation in wildlife trades and close contact with animal parts expose humans to contracting viral infections.

The majority of the respondents (98.4%) reported that COVID-19 outbreaks among the human population have occurred in Nigeria though (23.4%) reported to have seen humans with clinical symptoms indicative of COVID-19. Three-quarter of the animal health workers (74.9%) reported that companion animals can contract COVID-19 virus infection. Of the 336 responding to the question on which companion animal is most susceptible to and could spread the COVID-19 virus, 53.3% and 38.4% indicated that cats and dogs were the most susceptible, respectively. These findings suggest that animal health personnel responsible for the diagnosis and treatment of diseased animals should exercise more care when handling cases. However, only a few of the respondents (5.6%) reported having ever seen animals with clinical signs suggestive of COVID-19 infection. This is in line with what was reported by Cui *et al*.(2019) and Anderson *et al*.(2020) that coronaviruses have a broad horizon with multiple species of animals harboring these viruses, and simply a few of them get infected severely [38,39]. Recent studies have shown that the wide host range associated with the emergence of CoVs could be linked to the flux of the replicase enzyme RNA dependent RNA polymerase, polybasic furin cleavage site, and O-linked glycans, absence of proof-reading mechanism, a greater degree of transmutations in the RBD of spike gene and genetic recombination [40-42].

The majority of AHPs reported that COVID-19 can be transmitted as a form of zoonosis from animals to humans (68.1%) and as zoo-anthroponosis from humans to animals (66.3%). However, both forms of transmission have not been reported in Nigeria. The common mode of transmission is human to human due to usual contacts to droplets from cough or sneeze, overlays of public transport, cafes, eateries, and bars, and other public habitations of large meetings. This study reported that visiting live animal markets is of high zoonotic risk to the spread of coronavirus. The transfer of infectious diseases in live animal markets is high due to significant stress compromising the animals' immune systems and because of the number of different species being maintained near one another. An average percentage (52.0%) of the respondents perceived that the consumption of infected raw meat and meat products is of high zoonotic risk pathway to the transmission of COVID-19. However, most of the respondents to this survey (82.9% and 87.1%) regarded contact with infected animals and contaminated fomites as well as environmental contamination with viral aerosols to be linked with high zoonotic risk pathway for the spread of COVID-19.

A high proportion of respondents (98.4%) to this survey reported proper personal hygiene, work practices, and animal handling protocols using PPE when handling suspected animals, and sterilizing all equipment after usage as mitigation practices they engage in COVID-19 control. It is higher than reported in the study carried out in Ghana where 89.4% washed their hands with soap, 72.3% used hand sanitizers to decontaminate their hands [32]. The preparedness level could be as a result of the respondents´ awareness regarding the high infectivity level of COVID-19 which spread through respiratory droplets. Age, marital status, professional status, and geopolitical zone of respondents were significant with satisfactory knowledge of COVID-19. This is in congruence with the study carried out by Dryhurst *et al*. [43]. The association between socio-demography showed that high level of knowledge in the age category of 26-45 years and especially among the males can be attributed to the normal expectancy of males to be in the forefront of tasking events, ease of accessibility to various media through the internet [28] and the fact that this age group has more future active years, thus the need to stay healthy [31,44].

Our findings that veterinarians were more expected to possess adequate knowledge about COVID-19 and its necessary mitigation strategies are predictable. This is also consistent with an earlier report where veterinarians demonstrated appreciable knowledge of zoonotic diseases, risk perceptions, and preventive practices among the AHPs in Nigeria [23]. The Food and Agriculture Organization (FAO) states that physical and mental health issues and increased pressure and stress are of major concern for individuals working in the livestock value chain (farmers and AHPs) when confronted with COVID-19 and recommends measures to mitigate its risk [45]. It is thereby imperative to state that one's level of knowledge about an infectious disease can make one behave in ways that mitigate its spread [44] which is true of the average veterinarian as compared to the level of satisfactory knowledge observed among other animal health care providers. The level of awareness of these workers, especially the para-veterinarians and technologists, needs to be improved. The main limitation of this survey was that the data was collected by electronic distribution of the survey tool through the social media outlets with only individuals using the internet partaking in the study. However, we ensured that the desired sampled size was met and workers from all geo-political zones in the country were sampled. This could be representative of the limited internet users.

## Conclusion

Overall, participants in this survey have an appreciable knowledge level of COVID-19 and good mitigation measures instituted while working at their stations. However, our results still demonstrate the need to increase the level of awareness of para-veterinarians and technologists in the animal health sector in Nigeria. Furthermore, to control the disease, there is the need to enlighten the respondents more about the various zoonotic risk pathways to the spread of the disease and other preventive preparedness strategies especially with a more devastating second wave of the pandemic experienced across the country.

### What is known about this topic

- COVID-19 constitutes a threat to humans since most apparently healthy animals may potentially infect and cause the disease in humans, especially animal health professionals;
- The One Health concept is useful in understanding the spread, prevention, and control of COVID-19.

### What this study adds

- The majority of the surveyed animal health professionals have satisfactory knowledge about COVID-19;
- Age, marital status, professional status, and geopolitical zone were significantly associated with knowledge of COVID-19 among respondents;
- Animal health professionals have a high-risk perception on zoonotic pathways of the spread of COVID-19.
